# Study of a Kawasaki disease diagnostic prediction model based on the LightGBM machine learning algorithm

**DOI:** 10.3389/frai.2026.1776007

**Published:** 2026-05-25

**Authors:** Hongyan Li, Yushan Li, Chuxiong Gong, Ying Xiao, Zhongjian Su, Linfei Han, Hong Li, LiLi Deng, Bin Li, Yanfei Chen, Xing Zhang

**Affiliations:** 1 Department of Cardiology, Kunming Children’s Hospital, Kunming, Yunnan, China; 2 Department of Science and Education, Kunming Children’s Hospital, Kunming, Yunnan, China

**Keywords:** diagnostic model, Kawasaki disease, LightGBM, machine learning, SHAP

## Abstract

**Background:**

Kawasaki disease (KD) is an acute systemic vasculitis predominantly affecting children under 5 years of age. Its pathogenesis remains incompletely understood, and the lack of specific diagnostic biomarkers during the acute phase poses substantial challenges to clinical diagnosis. Such diagnostic uncertainty often results in missed or delayed cases, leading to lost therapeutic opportunities and the subsequent development of coronary artery lesions (CAL). The present study aimed to establish a machine learning–based diagnostic model to optimize the KD diagnostic workflow, enable early identification, and reduce the incidence of CAL.

**Methods:**

We retrospectively analyzed Pre-treatment clinical data from 4,469 patients admitted to Kunming Children’s Hospital between January 2017 and December 2023, including 2,345 patients diagnosed with Kawasaki disease (case group) and 2,124 febrile non-KD patients (control group). A Light Gradient Boosting Machine (LightGBM) algorithm was employed to develop a diagnostic model for KD. Model performance was assessed using the area under the receiver operating characteristic curve (AUC), as well as accuracy, sensitivity, and specificity calculated from the confusion matrix at a classification threshold of 0.5. SHapley Additive exPlanations (SHAP) analysis was performed to identify key predictive features and interpret the model’s decision-making mechanism. On the basis of SHAP-derived feature importance and clinical availability, six core variables were selected to establish a simplified diagnostic model, which was further deployed as an offline application.

**Results:**

The full-feature LightGBM model achieved an outstanding area under the receiver operating characteristic curve (AUC) of 0.9956. At a classification threshold of 0.5, the confusion matrix yielded an accuracy of 0.9653, a sensitivity of 0.9596, and a specificity of 0.9717. SHAP analysis revealed that C-reactive protein (CRP), activated partial thromboplastin time (APTT), total calcium (Ca^2+^), erythrocyte sedimentation rate (ESR), serum chloride (Cl^−^), and several other variables exhibited strong predictive value. To achieve an optimal balance between predictive performance and clinical applicability, six core variables were selected: white blood cell count (WBC), platelet count (PLT), CRP, APTT, thrombin time (TT), and albumin (ALB). The simplified LightGBM model constructed using these parameters achieved an AUC of 0.9792; at the 0.5 classification threshold, it demonstrated an accuracy of 0.9340, a sensitivity of 0.9277, and a specificity of 0.9410, indicating robust discriminative capacity. Finally, an offline executable application was developed to support clinicians—especially those in primary care settings—in achieving rapid and accurate early diagnosis of KD.

**Conclusion:**

Machine learning approaches enable effective simplification of the diagnostic process for KD. The predictive model constructed on the basis of WBC, PLT, CRP, APTT, TT, and ALB shows high diagnostic efficacy and favorable clinical applicability.

## Introduction

Kawasaki disease (KD), also known as mucocutaneous lymph node syndrome (MCLS), is most prevalent among children of Asian descent, typically affecting infants and children aged 6 months to 5 years (Kawasaki, 1967). The primary pathological alteration involves systemic inflammation of small- and medium-sized vessels, with coronary artery lesions (CAL) representing the principal complication (Jone et al., 2024; Wang et al., 2023). KD has emerged as a leading cause of acquired heart disease in children (Kawasaki, 1967). Without timely intervention, the risk of coronary artery damage is approximately 25% (La Vecchia et al., 2024). Intravenous immunoglobulin (IVIG) is globally recognized as an effective therapy for reducing cardiovascular events; early diagnosis followed by prompt IVIG treatment can decrease the incidence of CAL to approximately 3% (Newburger et al., 1991; Nait-Ladjemil et al., 2025).

Standardized diagnostic criteria for Kawasaki disease (KD) have been established (Jone et al., 2024), Complete KD diagnosis requires fever plus at least four of the following clinical features: (1) polymorphous rash; (2) bilateral bulbar conjunctival injection without exudate; (3) oral mucosal changes; (4) extremity changes; and (5) cervical lymphadenopathy (diameter ≥ 1.5 cm). Incomplete KD (IKD) is defined in patients with persistent fever for ≥ 5 days who fulfill only 2–3 of the above clinical criteria. Alternatively, for infants with fever lasting ≥ 7 days that cannot be explained by other diseases, a diagnosis of IKD may be considered if C-reactive protein (CRP) ≥ 30.0 mg/L and/or erythrocyte sedimentation rate (ESR) ≥ 40 mm/h, accompanied by three or more of the following abnormal laboratory findings: (1) white blood cell (WBC) count ≥ 15 × 10^9^/L; (2) platelet count (PLT) ≥ 450 × 10^9^/L after 7 days of fever; (3) mild to moderate anemia; (4) albumin≤ 30.0 g/L; (5) elevated alanine aminotransferase (ALT) (6) urinary WBC ≥ 10 per high-power field, or positive echocardiographic findings. Despite these guidelines, approximately 19.4% of IKD patients—particularly infants with atypical presentations—are missed or misdiagnosed (Tse et al., 2023). Even with standardized treatment, missing the optimal therapeutic window can lead to CAL in roughly 85% of delayed cases. Currently, there is no “gold standard” for KD diagnosis. Typical clinical manifestations are non-specific, and the assessment of clinical signs is often subjectiveFurthermore, the diagnosis of IKD relies on multiple laboratory tests that may be difficult to access in primary hospitals. Urine sampling in infants and young children is technically difficult and frequently contaminated, potentially resulting in laboratory errors. These factors hinder early recognition, leading to delayed diagnoses and subsequent adverse outcomes. Therefore, there is an urgent clinical demand for a simple, feasible diagnostic prediction model with high sensitivity and specificity to enable early and rapid identification of KD, thereby reducing the occurrence of CAL and improving the long-term prognosis of affected children.

Machine learning (ML), a branch of artificial intelligence, allows algorithms to recognize patterns within large datasets and generate accurate predictive outcomes (Broderick et al., 2023). Although ML has been widely applied in disease diagnosis and prediction, and is also extensively used in pediatrics, it has been rarely applied in KD (Baştanlar and Ozuysal, 2013; Deo, 2015; Helguera-Repetto et al., 2020; Lin et al., 2022; Byeon, 2020; Argalious and Farag, 2020). Existing diagnostic models for KD have demonstrated promising performance; however, they frequently rely on low-specificity features (e.g., subjective clinical signs) or indicators with limited availability, which impedes their practical application in primary clinical settings (Can Demirbaş et al., 2024; Kobayashi et al., 2020). Consequently, there remains a critical shortage of predictive models that effectively balance high diagnostic performance with clinical feasibility.

Light Gradient Boosting Machine (LightGBM) is a decision-tree-based gradient boosting framework characterized by high efficiency, memory economy, and high accuracy. It has been increasingly utilized in large-scale medical research (Duan et al., 2025; Zheng et al., 2025; Ke et al., 2017; Liao et al., 2022). SHAP (SHapley Additive exPlanations) is a robust interpretability tool that quantifies the contribution of each feature to the model’s predictions (Wang et al., 2022; Chowdhury et al., 2022). In this study, we developed a LightGBM-based diagnostic model to provide rapid and accurate early diagnosis of Kawasaki disease (KD). We further employed SHAP to elucidate the influence of clinical features on the model’s diagnostic performance and deployed a simplified offline model to enhance its clinical applicability.

In the present study, we developed a LightGBM-based machine learning diagnostic model to enable fast and accurate early diagnosis of Kawasaki disease (KD) and employed SHAP (SHapley Additive exPlanations) to analyze the influence of clinical features on KD prediction. To enhance the practicality and clinical applicability of the model, we utilized visualization techniques and deployed the final simplified model as an offline auxiliary program for KD diagnosis.

## Materials and methods

### Study design and overall workflow

This study aimed to develop an interpretable ML model to assist in the rapid differentiation of KD from other febrile illnesses. The study design comprised five stages: (1) patient selection and data collection; (2) data preprocessing and variable harmonization; (3) feature selection and model training; (4) model evaluation and interpretation; (5) model visualization and deployment.

### Study population

This study was a retrospective study, and the original data were extracted from the electronic medical record system of Kunming Children’s Hospital, including 2,345 children with acute KD in the experimental group and 2,124 children with infectious fever in the control group (CG) who met the inclusion and exclusion criteria from January 2017 to December 2023. All data were the laboratory indicators of the patients after hospitalization but before treatment. Inclusion criteria for KD: (1) Diagnosis conforming to the American Heart Association (AHA) 2024 Scientific Statement (Jone et al., 2024); (2) First-time KD diagnosis with hospitalization on day 5–9 of fever. Exclusion criteria for KD: Congenital or valvular heart disease, myocardial infarction, arrhythmia, cardiomyopathy, pulmonary hypertension, or hypertensive heart disease; presence of other infectious, immune, or hematologic diseases; digestive system diseases; pre-admission medications affecting hematologic parameters; subacute or recovery phase KD at presentation; and specific febrile illnesses such as typhoid, scarlet fever, or sepsis. Control group criteria: Diagnosis of infectious fever, completion of therapy, and complete baseline data. Patients with concomitant hematologic malignancies or neoplastic diseases were excluded. The study was approved by the Ethics Committee of Kunming Children’s Hospital (Approval no.: 2022-02-212-KO1).

Modeling proceeded in two rounds. In round one, all available laboratory indices were used to train a full-feature model and SHAP was used to evaluate variable contributions. In round two, guided by clinicians, six widely available and representative variables were selected to construct a simplified model balancing predictive performance and clinical applicability. The final model was packaged as an offline executable suitable for primary hospitals with limited network connectivity.

### Data collection and preprocessing

The dataset was randomly stratified into a training set 80%, *n* = 3,575 and a test set 20%, *n* = 894. Systematic data cleaning was performed; only features present in both groups were retained. Variables with >25% missing values were excluded. Ultimately, 51 clinical indicators were included for initial model construction (Table 1). To manage extreme values in key variables, reasonable upper and lower bounds were established (truncation): CRP values < 0.5 mg/L or > 200 mg/L were set to 0.5 and 200, respectively; PCT < 0.25 or > 100 were set to 0.25 and 100. Gender was numerically encoded (male = 0, female = 1). For machine-learning model development, missing values in continuous variables were imputed using the median via sklearn.impute. SimpleImputer in Python.

**Table 1:** Baseline characteristics of the KD and CG groups.

Variable	KD (*n =* 2,345)	CG (*n =* 2,124)	*P*-value	Sig
Month-old	23.0 [12.0, 39.0]	39.0 [18.0, 69.0]	<0.001	***
Male, *n* (%)	1,460 (62.3%)	1,250 (58.9%)	0.022	*
WBC, 10^9/L	12.8 [9.5, 16.6]	6.8 [4.5, 9.8]	<0.001	***
PLT, 10^9/L	393.0 [304.0, 504.0]	288.0 [209.0, 382.0]	<0.001	***
Red Blood Cell (RBC), 10^12/L	4.2 [3.9, 4.5]	4.6 [4.0, 4.9]	<0.001	***
Hemoglobin (Hb), g/L	111.0 [103.0, 119.0]	122.0 [108.0, 132.0]	<0.001	***
Mean Corpuscular Volume (MCV), fL	80.0 [77.1, 82.6]	81.3 [78.0, 85.1]	<0.001	***
Mean Corpuscular Hemoglobin (MCH), pg	26.8 [25.5, 27.8]	27.4 [26.2, 28.7]	<0.001	***
Mean Corpuscular Hemoglobin Concentration (MCHC), g/L	333.0 [325.0, 341.0]	334.0 [325.0, 343.0]	0.005	**
Hematocrit (HCT), %	33.3 [31.1, 35.6]	36.4 [32.8, 39.1]	<0.001	***
Red Cell Distribution Width - Coefficient of Variation (RDW-CV), %	13.5 [12.9, 14.2]	13.6 [12.9, 15.0]	<0.001	***
Red Cell Distribution Width - Standard Deviation (RDW-SD), fL	39.2 [37.4, 41.3]	39.9 [37.7, 43.3]	<0.001	***
Mean Platelet Volume (MPV), fL	9.2 [8.6, 9.8]	9.5 [8.9, 10.2]	<0.001	***
Platelet Distribution Width (PDW), fL	9.9 [9.1, 11.1]	10.5 [9.6, 11.8]	<0.001	***
PCT, %	0.4 [0.3, 0.5]	0.3 [0.2, 0.4]	<0.001	***
Platelet Large Cell Ratio (P-LCR), %	17.8 [13.6, 23.2]	20.4 [16.2, 26.0]	<0.001	***
Lymphocyte count, 10^9/L	3.5 [2.4, 5.0]	2.8 [1.7, 4.4]	<0.001	***
Neutrophil count, 10^9/L	7.8 [4.8, 10.9]	2.5 [1.2, 4.7]	<0.001	***
Monocyte count, 10^9/L	0.7 [0.5, 1.1]	0.5 [0.3, 0.8]	<0.001	***
Lymphocyte %, %	28.9 [19.1, 41.2]	46.1 [31.1, 61.9]	<0.001	***
Neutrophil %, %	60.8 [46.4, 72.7]	40.3 [25.0, 57.0]	<0.001	***
ESR, mm/h	63.0 [45.0, 83.0]	23.0 [12.0, 38.0]	<0.001	***
CRP, mg/L	66.3 [29.0, 116.6]	6.1 [0.9, 22.3]	<0.001	***
Total bilirubin, μmol/L	8.1 [5.9, 11.5]	8.8 [6.7, 11.8]	<0.001	***
Direct bilirubin, μmol/L	3.0 [2.2, 4.6]	3.0 [2.3, 4.2]	0.523	
Indirect bilirubin, μmol/L	4.9 [3.2, 7.0]	5.7 [4.2, 8.0]	<0.001	***
Total protein, g/L	60.1 [55.2, 65.4]	66.0 [61.5, 70.3]	<0.001	***
Albumin, g/L	33.4 [30.2, 36.5]	39.2 [35.8, 42.2]	<0.001	***
Globulin, g/L	25.9 [22.7, 30.1]	26.6 [22.9, 30.4]	0.229	
Alanine AminotransferaseALT, U/L	27.0 [15.0, 64.0]	18.0 [13.0, 27.0]	<0.001	***
Aspartate Aminotransferase (AST), U/L	31.0 [24.0, 44.0]	36.0 [28.0, 48.0]	<0.001	***
Alkaline Phosphatase (ALP), U/L	171.0 [139.0, 213.0]	183.0 [147.0, 230.0]	<0.001	***
Gamma-Glutamyl Transferase (GGT), U/L	38.0 [16.0, 100.0]	13.0 [11.0, 19.0]	<0.001	***
Total bile acid (TBA), μmol/L	7.5 [4.6, 12.0]	6.1 [3.3, 10.4]	<0.001	***
Urea, mmol/L	3.0 [2.3, 3.9]	3.7 [2.9, 4.7]	<0.001	***
Creatinine (Cr), μmol/L	19.0 [15.0, 24.0]	24.0 [19.0, 31.0]	<0.001	***
Uric acid (UA), μmol/L	214.2 [169.0, 269.3]	256.0 [206.0, 313.6]	<0.001	***
(Potassium) K^+^, mmol/L	4.4 [3.9, 4.8]	4.3 [4.0, 4.7]	0.170	
Sodium (Na^+^), mmol/L	136.0 [134.0, 138.0]	139.0 [137.0, 141.0]	<0.001	***
Chlorine (Cl^−^), mmol/L	100.0 [97.0, 103.0]	104.0 [102.0, 106.0]	<0.001	***
Magnesium (Mg^2+^), mmol/L	0.9 [0.8, 0.9]	0.9 [0.8, 1.0]	<0.001	***
Pi, mmol/L	1.4 [1.2, 1.6]	1.5 [1.4, 1.7]	<0.001	***
Total (Ca^2+^), mmol/L	2.2 [2.1, 2.4]	2.4 [2.3, 2.5]	<0.001	***
Creatine Kinase-Myocardial Band (CK-MB), μg/L	16.0 [13.0, 21.7]	19.0 [14.0, 25.0]	<0.001	***
IgA, g/L	0.6 [0.4, 1.0]	0.6 [0.3, 1.0]	0.338	
IgM, g/L	1.1 [0.8, 1.5]	0.9 [0.6, 1.3]	<0.001	***
IgG, g/L	6.3 [4.7, 8.6]	6.9 [5.2, 8.8]	<0.001	***
PT, s	12.5 [11.6, 13.4]	12.6 [12.0, 13.3]	<0.001	***
APTT, s	32.5 [27.5, 37.9]	39.8 [36.1, 43.8]	<0.001	***
Fibrinogen, g/L	5.4 [4.5, 6.1]	3.3 [2.6, 4.2]	<0.001	***
TT, s	16.1 [15.0, 17.5]	17.5 [16.6, 18.5]	<0.001	***

Data are presented as median (IQR) or *n* (%). Continuous variables were compared using the Mann–Whitney U test, and categorical variables were compared using the chi-square test. Baseline comparisons in the table were performed using the original observed data rather than imputed values. HC, healthy control; KD, Kawasaki disease. Sig: *p < 0.05, **p < 0.01, ***p < 0.001. Gender was coded as male = 0 and female = 1.

### Statistical analysis

Baseline comparisons in Table 1 were performed using the original observed data rather than imputed values. Continuous variables were assessed according to their distribution characteristics. Because all continuous variables showed non-normal or skewed distributions in at least one group, they are presented as median (interquartile range, IQR) and were compared using the Mann–Whitney U test. Categorical variables are presented as *n* (%) and were compared using the chi-square test. A two-sided *p* < 0.05 was considered statistically significant.

For model evaluation on the independent test set, in addition to AUC and accuracy, sensitivity and specificity were calculated from the confusion matrix at a classification threshold of 0.5.

### Model construction and interpretation

Model development proceeded in two phases. First, a full-feature model was trained using all processed indicators, followed by interpretation using SHAP. Second, to improve operability in primary care settings, a simplified model was developed using six clinically accessible, high-impact features identified by SHAP. LightGBM was employed for both models. Model evaluation was assessed on the independent test set using the AUC, accuracy, and sensitivity and specificity derived from the confusion matrix at a classification threshold of 0.5 on the independent test set.

### Application development

To enhance clinical utility, we developed an offline diagnostic tool: a Windows-compatible executable (.exe) embedding the simplified LightGBM model. This tool provides an intuitive interface for clinicians to input the six laboratory values and receive a probabilistic KD prediction.

## Results

### Baseline characteristics

The baseline characteristics of the KD and CG are summarized in Table 1. Compared with CG, patients with KD were younger and showed significantly higher levels of WBC, PLT, CRP, ESR, neutrophil count, fibrinogen, and Gamma-Glutamyl Transferase (GGT), together with significantly lower levels of ALB, sodium (Na^+^), chloride (Cl^+^), total calcium (Ca^2+^), APTT, and TT. Most laboratory variables differed significantly between groups, whereas direct bilirubin (Tibl), globulin (GLB), potassium (K+), and Immunoglobulin A (IgA) did not show significant between-group differences.

### Full-feature model performance

**Figure 1:**
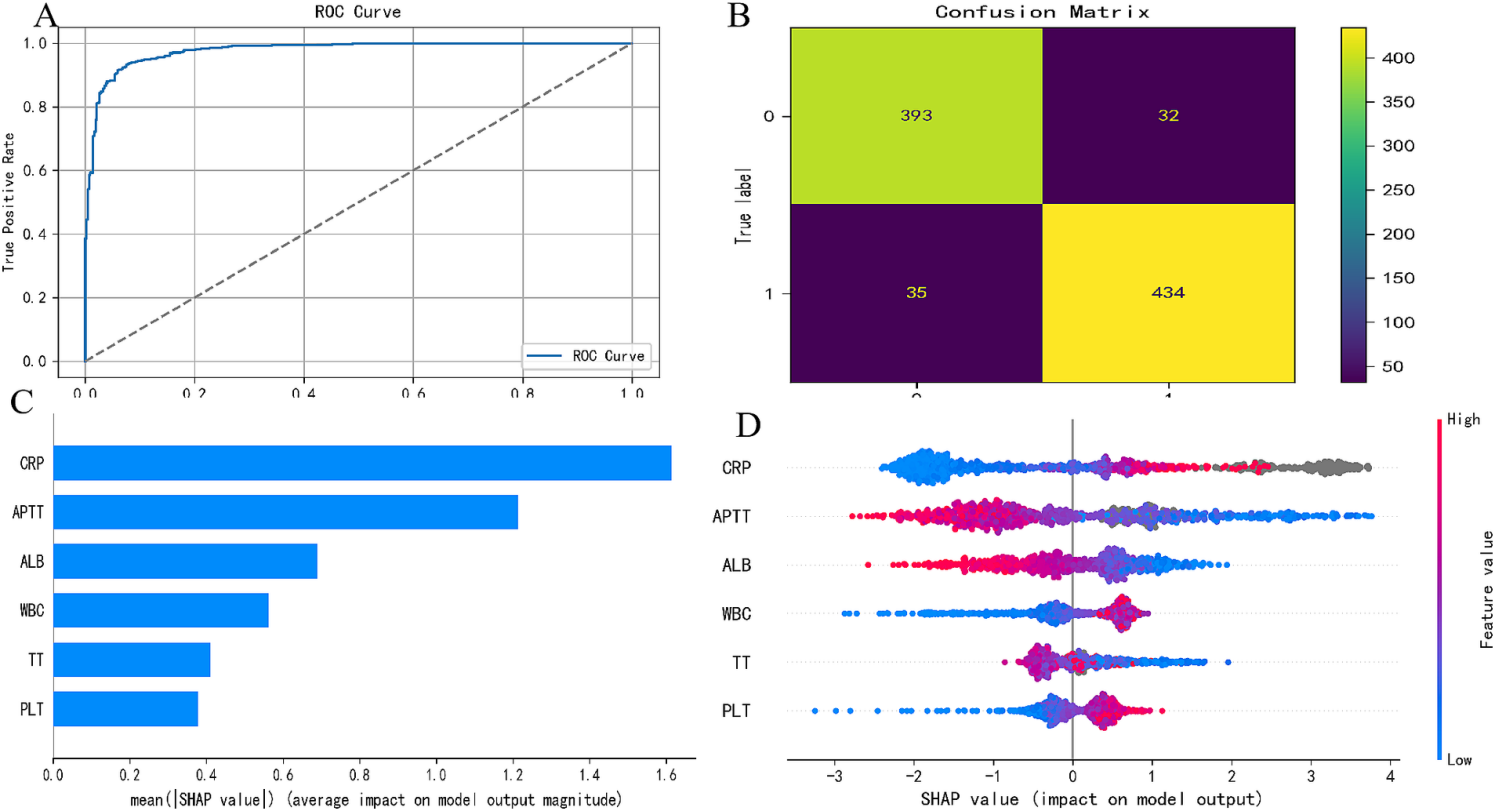
**(A)** ROC curve. **(B)** Confusion matrix. **(C)** Bar chart of mean SHAP importance (features ranked by mean absolute Shapley value). **(D)** SHAP summary dot plot for all features, horizontal axis is SHAP value, and features are ordered by mean absolute SHAP.

In the initial modeling stage, the full-feature model exhibited excellent discriminative ability on the independent test set, achieving an ROC AUC of 0.9956 (Figure 1A). At the classification threshold of 0.5, the confusion matrix showed an accuracy of 0.9653, a sensitivity of 0.9596, and a specificity of 0.9717 (Figure 1B). SHAP analysis provided model interpretability (Figures 1C,D); the model responded strongly to several features, particularly age, WBC, CRP, ALB, TT, and APTT.

### Simplified model and performance comparison

Based on SHAP importance and clinical availability, six core variables were selected: WBC, PLT, CRP, APTT, TT, and ALB. This simplification enhanced operability for primary care without substantially compromising performance. While the full - feature model achieved the highest accuracy, its requirement for numerous inputs limits its utility in resource - constrained settings.

**Figure 2:**
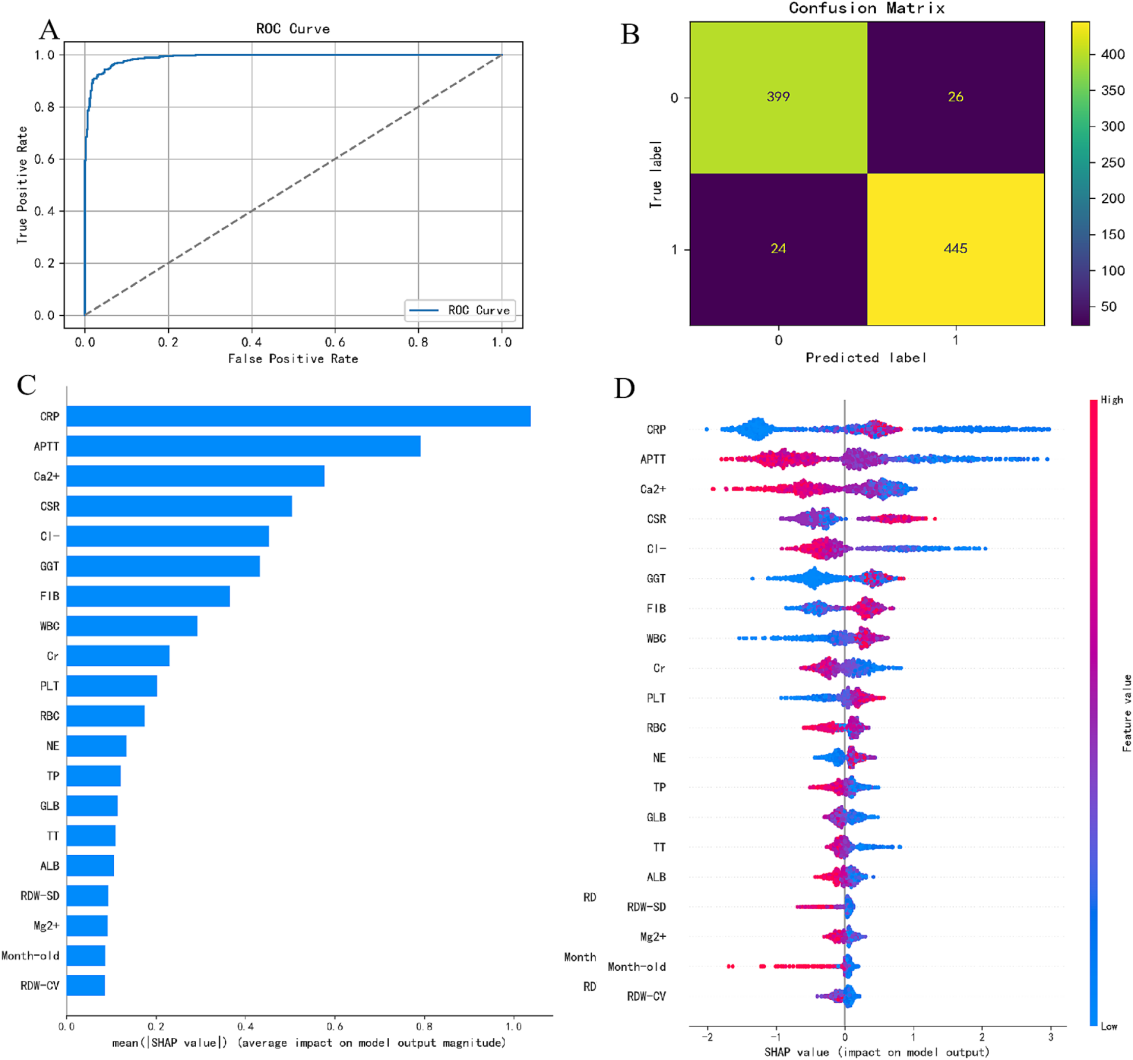
**(A)** ROC curve. **(B)** Confusion matrix, illustrating balanced performance across KD and CG. **(C)** Mean SHAP summary bar chart. **(D)** SHAP summary dot plot, showing each variable’s impact and directionality across samples.

The simplified model maintained robust predictive performance on the independent test set, with an ROC AUC of 0.9792 (Figure 2A). At the classification threshold of 0.5, the confusion matrix showed an accuracy of 0.9340, a sensitivity of 0.9277, and a specificity of 0.9410 (Figure 2B). SHAP analysis of the simplified model confirmed the contributions of the six variables (Figures 2C,D), identifying CRP, APTT, and ALB as the primary drivers. This highlights the critical role of inflammatory and coagulation - related indices in early KD identification.

### Software development and model deployment

**Figure 3:**
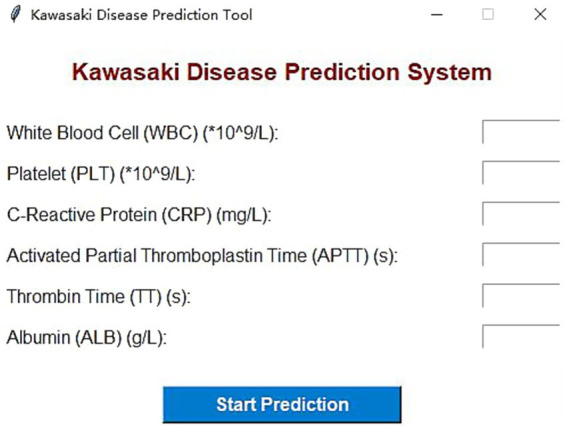
KD offline diagnosis program operation interface.

The six-feature LightGBM prediction model was packaged as a standalone Windows executable (.exe) with a graphical user interface (GUI), enabling KD risk assessment without network connectivity or a local Python environment. Developed using Python 3.10 and tkinter (packaged via Nuitka), the software allows users to input six values (WBC, PLT, CRP, APTT, TT, and ALB) to obtain a one-click probability prediction (Figure 3). This tool is designed to assist early risk assessment in settings with limited resources.

## Discussion

KD is a systemic vasculitis predominantly affecting children, which can lead to coronary artery aneurysms and represents a leading cause of acquired heart disease in the pediatric population (Kawasaki, 1967). Current diagnostic approaches rely primarily on clinical manifestations and lack specific quantitative biomarkers; Notably, IKD also need to be diagnosed with the assistance of laboratory indicators. However, some laboratory test indicators (such as infant urine specimens) are difficult to collect, and primary hospitals often face challenges in accessing these indicators. This further highlights the urgency of developing a simple, easily implementable diagnostic model that maintains high diagnostic performance. Although ML has been increasingly applied in medical diagnostics, studies leveraging large-scale datasets for KD prediction remain relatively limited. The principal contribution of this study is the development of a LightGBM-based, interpretable diagnostic model for KD, validated by SHAP visualization. We elucidated the internal decision logic and identified clinical features with the most significant influence on predictions. Crucially, we addressed the challenge of IKD diagnosis by developing an offline application based on six common tests, thereby improving clinical applicability.

SHAP analysis identified CRP as the strongest predictor, consistent with the acute systemic inflammatory nature of KD (Kawasaki, 1967). However, as CRP lacks specificity (Philip et al., 2023; Martini et al., 2022), our model integrates it with other features (hematologic and coagulation indices) via LightGBM to generate a more precise composite assessment. This demonstrates the capacity of ML to synthesize multidimensional information for complex diagnostic tasks.

APTT was identified as one of the most important predictors in the model. Its contribution should be interpreted in the context of multivariable interactions captured by LightGBM, rather than solely according to univariable between-group differences. This may reflect the complex coagulation-related alterations associated with KD (Menon et al., 2022). Notably, SHAP indicated that total Ca^2+^ ranked highly (third) and was negatively associated with KD prediction—an influence exceeding that of traditional markers such as WBC, PLT, ALB, or ALT (Kawasaki, 1967). The underlying mechanisms may include: (1) acute systemic inflammation and impaired hepatic synthetic function in KD leading to hypoalbuminemia, and because about half of serum calcium is albumin-bound, reduced albumin lowers measured total calcium (Vergara et al., 2021); (2) mounting evidence that calcium ions play roles in immune regulation and endothelial function—intracellular calcium signaling is key to lymphocyte activation and inflammatory mediator release; hypocalcemia may modulate immune cell activity and inflammation magnitude (Desgagnés et al., 2025); (3) calcium’s role in maintaining endothelial barrier integrity and vascular tone, where severe hypocalcemia could increase vascular permeability and exacerbate edema and inflammation (Behin et al., 2003). Therefore, the strong association between low total calcium and KD risk may reflect a combination of hypoalbuminemia, immune dysregulation, and endothelial dysfunction. Clinically, assessment of calcium metabolism may be valuable in KD workup. Future research should clarify causal relationships among total calcium, ionized calcium, KD, and coronary artery lesions and explore whether correcting hypocalcemia could improve outcomes.

Traditional KD diagnostic prediction studies have frequently used logistic regression due to its simplicity and interpretability; however, logistic models may struggle with nonlinear, high-dimensional, multi-center large datasets and cannot easily capture interactions among variables, limiting performance. Machine learning methods can overcome these limitations. Qu et al. (2025) (from 3,650 patients: 2,299 KD and 1,351 similar non-KD) collected 43 clinical features and tested 10 machine learning algorithms; XGBoost performed best with AUC = 0.9833. Relying on the top 10 features the model AUC was 0.9757 and a web diagnostic tool was developed. Duan et al. (2025) used Xtreme Gradient Boosting on 1,089 KD and 81,697 non-KD febrile children with 25 hematology indices to build a KD diagnostic model achieving AUC = 0.9999. In our study, the LightGBM full-feature model achieved AUC = 0.9956, comparable to prior work while using a richer set of clinical features. Balancing sample size and model performance, we selected six clinically accessible core features (WBC, PLT, CRP, APTT, TT, and ALB) deployed the simplified model as an offline program (AUC = 0.9792), offering clear advantages in discriminative ability and operationalizability.

In summary, SHAP-guided interpretation confirms that the LightGBM KD prediction model’s decision logic aligns with existing medical knowledge and may reveal novel candidate biomarkers (e.g., calcium, chloride, magnesium). The model integrates demographics, inflammatory markers, coagulation, and biochemical data, demonstrating potential as a clinical decision support tool. Nonetheless, this study has several limitations. First, it was a retrospective single-center study based on data from Kunming Children’s Hospital, which may limit the generalizability of the model to other institutions, regions, or patient populations. Although we performed internal validation using an independent held-out test set, this does not replace true external validation. Therefore, future work should include multicenter external validation using independent cohorts from different hospitals and clinical settings to further assess model robustness and transportability.

In conclusion, we successfully developed an interpretable machine learning-assisted diagnostic model for KD, which was constructed using six clinically easily accessible laboratory indicators (WBC, PLT, CRP, APTT, TT, ALB). This model not only exhibits high clinical operability but also possesses excellent diagnostic efficacy, and it is presented in the form of an offline program. The model demonstrates providing rapid, intuitive decision support for clinicians. This tool holds the potential to improve long-term outcomes for KD patients and offers new avenues for research into KD pathophysiology.

## Data Availability

The raw data supporting the conclusions of this article will be made available by the authors, without undue reservation.
